# Human Intestinal Spirochetosis as a Cause of Chronic Diarrhea in an Immunocompetent Patient: A Case Report

**DOI:** 10.7759/cureus.100827

**Published:** 2026-01-05

**Authors:** Bi Zhang, Daniel Najar, Barry Li, David Suster, Weizheng Wang

**Affiliations:** 1 Department of Internal Medicine, Northwell Health, Bay Shore, USA; 2 Department of Internal Medicine, Rutgers University New Jersey Medical School, Newark, USA; 3 Department of Pathology, Immunology and Laboratory Medicine, Rutgers University New Jersey Medical School, Newark, USA; 4 Division of Gastroenterology and Advanced Endoscopy, Rutgers University New Jersey Medical School, Newark, USA

**Keywords:** chronic diarrhea, his, hiv, immunocompetent, weight loss

## Abstract

Human intestinal spirochetosis (HIS), commonly due to *Brachyspira aalborgi* or *Brachyspira pilosicoli* infection, represents the adhesion and colonization of spirochetes to the apical membrane of colonic and rectal epithelial cells. The clinical spectrum can range from asymptomatic to chronic watery diarrhea, abdominal pain, and, in rare cases, rectal bleeding. However, symptomatic cases are usually found in immunocompromised patients. It has been reported to have a high prevalence of human immunodeficiency virus (HIV) infection among homosexual men, cancer patients, and immunotherapy patients. It is also associated with irritable bowel syndrome, eosinophilic enterocolitis, possible colonic polyps, notable sessile serrated adenoma/polyp, and colon cancer. In this case report, an immunocompetent patient without risk factors presented with chronic watery diarrhea and unintentional weight loss of 3.18 kilograms after two months (body weight baseline was 74.5 kilograms, 4% of weight loss in two months). HIS was confirmed by biopsy through colonoscopy. The patient was treated with a 10-day course of metronidazole (500 mg, three times a day) with complete remission of diarrhea. Therefore, our study supports the necessity of considering HIS as part of the differential diagnosis for immunocompetent patients with chronic diarrhea and weight loss, despite risk factors.

## Introduction

Spirochetes are gram-negative, motile, spiral, or corkscrew bacteria with unique endocellular flagella. They are well-known for the etiology of human diseases such as Lyme disease, syphilis, or leptospirosis [1]. Human intestinal spirochetosis (HIS), first described in 1967, is the adhesion and colonization of spirochetes to the apical membrane of colonic and rectal epithelial cells [2]. The most common organisms are *Brachyspira aalborgi* or *Brachyspira pilosicoli*. The prevalence of HIS varies significantly by region and population. It ranges between 2% and 7% in developed countries and up to 34% in underdeveloped countries. The prevalence is even up to 54% in homosexual men and human immunodeficiency virus (HIV) -infected patients [3]. While HIS was usually considered to have few symptoms and antibiotic treatment is usually deferred for patients with normal immune status, symptomatic HIS infection is commonly seen in immunocompromised patients, such as those with HIV, cancer, or immunotherapy [4,5]. However, there have been increasing reports that symptomatic HIS infection presents in immunocompetent patients. Therefore, there has been debate as to whether this organism is pathogenic or simply a commensal organism to the human body [6]. The common clinical presentation includes nausea, vomiting, diarrhea, abdominal pain, weight loss, and blood-stained stool, with the transmission route being fecal-oral [7]. Here, we report a case of a 50-year-old male patient with normal immune status who presented with chronic diarrhea and a 3.18 kg unintentional weight loss after two months. His colonoscopy had grossly normal findings, yet with confirmed intestinal spirochetosis infection on biopsy.

## Case presentation

A 50-year-old man with a past medical history of chronic obstructive pulmonary disease (COPD), asthma, and hypertension presented to the gastroenterology clinic with two months of chronic watery diarrhea and unintentional weight loss of 3.18 kg (body weight baseline was 74.5 kg, 4% of weight loss in two months). He described 4-6 watery, soft bowel movements daily, denied hematochezia, nausea, vomiting, dysphagia, abdominal pain, or constipation. No greasy, oily, floating stools or other fat malabsorption features were reported. He denied recent antibiotic exposure, recent travel history, or sick contacts and also denied the history of inflammatory bowel disease (IBD) or immune suppressive medication use. He consumed approximately six beers daily and smoked a 20-pack-year. Family history was unremarkable. On arrival, the vitals and physical exam were normal. Labs included complete blood count with differential, comprehensive metabolic panel, and liver function tests. The evaluation was unremarkable, including the normal white blood cell count and mild microcytic anemia (Table 1).

**Table 1:** Laboratory values with reference ranges

Laboratory test	Parameter	Result	Normal range
	White blood cell	6.8 K/uL	3.80-10.50 K/uL
	Red blood cell	5.7 M/uL	4.20-5.80 M/uL
	Hemoglobin	13.5 g/dL	13.0-17.0 g/dL
	Hematocrit	42.9 %	39.0-50.0 %
	Mean corpuscular volume	75.2 fl	80.0-100.0 fl
	Mean corpuscular hemoglobin	23.7 pg	27.0-34.0 pg
Complete blood count	Mean corpuscular hemoglobin concentration	31.5 g/dL	32.0-36.0 g/dL
	Red cell distribution width	14.5%	10.3-14.5 %
	Platelet	339 K/uL	150-400 K/uL
	Mean platelet volume	7.5 fL	7.0-13.0 fL
	Auto neutrophils %	68.4%	43.0-77.0 %
	Auto lymphocytes%	19.2%	13.0-44.0 %
	Auto Monocytes %	8.2%	2.0-14.0 %
	Auto eosinophils %	3.3%	0.0-6.0 %
	Glucose	63 mg/dL	70-99 mg/dL
	Blood urea nitrogen (BUN)	10 mg/dL	8.0-20.0 mg/dL
	Sodium (Na)	140 mmol/L	135-145 mmol/L
	Potassium (K)	3.9 mmol/L	3.5-5.3 mmol/L
	Chloride	108 mmol/L	96-108 mmol/L
Comprehensive metabolic panel	Carbin dioxide	24 mmol/L	22.0-29.0 mmol/L
	Calcium	9.2 mg/dL	8.4-10.5 mg/dL
	Aspartate aminotransferase (AST)	20 U/L	<= 39 U/L
	Alanine aminotransferase (ALT)	16 U/L	<= 40 U/L
	Total protein	7.5 g/dL	6.6-8.7 g/dL
	Albumin	4.4 g/dL	3.3-5.2 g/dL
	Alkaline phosphatase	75 U/L	40-120 U/L
	Total bilirubin	0.4 mg/dL	0.4-2.0 mg/dL
	Creatinine	1.0 mg/dL	0.50-1.30 mg/dL
	Estimated glomerular filtration rate	92 ml/min/1.73m^2^	>=60 ml/min/1.73m^2^

**Figure 1:**
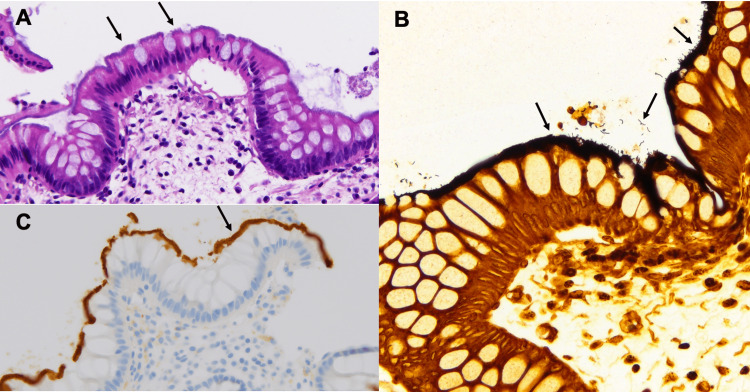
Histopathology of colon biopsies with stains revealing spirochetes adhered to the mucosa (A) Hematoxylin and eosin stain shows colonic mucosa lined by a thick layer of spirochetes creating a linear purple border along the surface (high-power magnification 40x). (B) Warthin-Starry special stain highlights the spirochetes in black. Notice a few spiral-shaped organisms breaking off from the surface layer (high-power magnification 40x). (C) *Treponema *immunohistochemistry shows intense brown staining along the surface of the mucosa with antibody against the spirochetes (high-power magnification 40x)

Of note, HIV antigen/antibody testing was nonreactive. Since the presence of alarm features, age over 50 years, unexplained weight loss, and diarrhea, the patient was prompted to undergo an early colonoscopy evaluation. Colonic biopsies revealed basophilic fringes along the epithelial surface on hematoxylin and eosin (H&E) staining, further accentuated by Warthin-Starry staining for spirochetes. Immunohistochemical analysis with an anti-*Treponema pallidum* antibody demonstrated intense brown staining along the mucosal surface, confirming the presence of spirochetes. Collectively, these histopathological findings are diagnostic of HIS (Figure 1).

The patient was subsequently treated with a 10-day course of metronidazole (500 mg, three times a day). At a follow-up appointment, he reported complete symptom resolution. Repeat colonoscopy was deferred, with standard colorectal cancer screening advised in 3-5 years.

In the United States, where resources are abundant, the common causes of chronic diarrhea include functional disorders such as irritable bowel syndrome (IBS); organic disorders such as IBD; malabsorption syndrome, including lactulose intolerance and celiac disease; and chronic infection, particularly in immunocompromised patients with persistent diarrhea. In our patient, the initial differential diagnoses included excessive alcohol intake, IBS, IBD, celiac disease, lactulose intolerance, or chronic infection such as giardiasis. The presence of alarm features warranted early colonoscopy evaluation. Histological findings indicated HIS infection, which is more commonly found in immunocompromised patients. The clinical manifestations of HIS are well-established and range from asymptomatic colonization to chronic watery diarrhea, abdominal pain, and, rarely, rectal bleeding. Symptomatic disease, however, is most frequently reported in immunocompromised patients.

## Discussion

The vast majority of current literature supports HIS diagnosis in homosexual men or immunocompromised patients in developed countries. Our patient, however, had significant symptoms associated with HIS despite having no risk factors. It should also be highlighted that, regardless of the diagnosis, our patient also presented with significant subacute to chronic symptoms. As for the literature, most current reports have found HIS in HIV patients, but our patient tested negative for HIV through standard HIV testing. Other risk factors for HIS include homosexual men, cancer patients, or immunotherapy patients, of which our patient did not fall under [6,8,9]. He also denied any history of immunosuppressive drug use or any medications, history of immunotherapies, and recent travel abroad, which further adds to the narrative that HIS is not just a disease in men who have sex with men populations and the immunocompromised.

The primary route of IS transmission is believed to be the fecal-oral route. Yet, the mechanism of infection is unclear. It is proposed that invasion beyond the surface epithelium is associated with initiating and developing symptoms and responding to antibiotic treatment, whereas patients with intact epithelium are likely asymptomatic [10]. Since spirochetes are difficult to grow in culture media or detect in stool examination, the gold standard of diagnosis of HIS is histological examination on the biopsy through colonoscopy. Histologically, it is characterized by a "fringe" or "carpet" of spirochetes adhesion and colonization on the apical membrane of colonic or rectal epithelial cells. The "fuzzy" or "basophilic" layer often appears as a "false brush border," which can be best visualized by Warthin-Starry or silver staining. More novel methods, such as polymerase chain reaction (PCR), fluorescent in situ hybridization (FISH), and immunomagnetic separation (IMS), were explored. Although those methods can specifically separate different spirochetal species and show promise for the future [11-14], morphological identification in intestinal biopsy is still the current gold standard for diagnosing HIS. Our patient had colonic mucosa lined by a thick layer of spirochetes on H&E stain, noticeable spirochetes on Warthin-Starry stain, and antibodies against the spirochetes on *Treponema *immunohistochemistry. Further molecular testing was deferred. 

The current relationship between a human host and HIS is thought to be commensal with normal gut flora, which is understood to have no significant underlying inflammation in patients with normal immune status, and antibiotic treatment is usually deferred [5]. Yet, its relationship to the human gastrointestinal tract is debated, whether this spirochate is actually pathogenic or not. Most cases in patients diagnosed with HIS without risk factors are asymptomatic, and diagnosis is usually found incidentally through routine biopsy and is thought to have no significant underlying inflammation in patients with normal immune status [4,5]. Christie et al. present the argument that IS is only clinically significant in certain cases but do not comment on what those cases are [15]. Similarly, Anthony et al. attempt to answer this question through a case series and suggest that pathologic HIS diagnosis depends on a variety of host and environmental factors [4]. These vague conclusions make it difficult to come to a concrete conclusion due to the small amount of literature on HIS. Our patient had significant symptoms, including weight loss, which contributed to our decision to treat him and a routine follow-up for further surveillance colonoscopy. In their meta-analysis, Fan et al. found a clear association between colonic spirochetosis and diarrhea, abdominal pain, and IBS [9]. These findings spark further debate as to whether HIS truly is a commensal organism and whether it should be treated even when asymptomatic patients with no risk factors present. Other literature found HIS to be associated with IBS, eosinophilic enterocolitis, possible colonic polyps, notable sessile serrated adenoma/polyp, and colon cancer [9,16-18]. Thus, these associations present the question of whether HIS is a downstream product of the other associated conditions or if HIS itself compromises the colonic mucosa, further leading to complications. Regardless, early identification of IS infection is crucial for all patients with similar symptoms.

As mentioned, the standard diagnosis of HIS is done through biopsy and is often found incidentally through routine colonic screening, as the affected mucosa often looks grossly normal. Anthony et al. again provided interesting statistics on HIS, where they found 5/26 (19%) of HIS cases in their database had endoscopic evidence of inflammation, and only 5/26 (19%) had the presence of inflammation on histologic sampling but had a confirmed diagnosis of HIS seen on the pathology report [4]. Therefore, HIS should always be on the differential in symptomatic patients despite gross endoscopic findings that appear normal. Much of the literature is in agreement that symptomatic patients warrant antimicrobial treatment, where many clinicians choose metronidazole as the drug of choice with high efficacy [18-20]. Hence, we decided to treat our patient with metronidazole, which resulted in the complete eradication of symptoms.

## Conclusions

This case is one of a few reports on a symptomatic HIS case in an immunocompetent individual with no other associated risk factors. HIS typically presents in immunocompromised individuals. In our patient, the presence of alarm features, age over 50, and diarrhea with unintentional weight loss prompted the early colonoscopy evaluation, which is consistent with standard clinical practice. Colonoscopy with biopsy revealed classic histopathologic findings diagnostic of HIS. The complete resolution of symptoms following metronidazole therapy supports the clinical significance of this finding. This study suggests that providers should consider HIS as part of the differential diagnosis when a patient presents with the above symptoms despite having no risk factors.
